# A new species of the genus *Acanthosaura* (Squamata, Agamidae) from Yunnan, China, with comments on its conservation status

**DOI:** 10.3897/zookeys.959.54601

**Published:** 2020-08-14

**Authors:** Shuo Liu, Mian Hou, Mingzhong Mo, Dingqi Rao

**Affiliations:** 1 Kunming Natural History Museum of Zoology, Kunming Institute of Zoology, Chinese Academy of Sciences, 32 Jiaochang Donglu, Kunming, Yunnan 650223, China; 2 College of Continuing (Online) Education, Sichuan Normal University, No. 5, Jing’an Road, Jinjiang District, Chengdu, Sichuan 610066, China; 3 Honghe Prefecture Forestry and Grassland Bureau of Yunnan Province, Honghe Avenue and Tianzhu Road’s intersection, Mengzi, Yunnan 661199, China; 4 Kunming Institute of Zoology, Chinese Academy of Sciences, 32 Jiaochang Donglu, Kunming, Yunnan 650223, China

**Keywords:** endangered, Honghe, lizard, taxonomy

## Abstract

A new species of *Acanthosaura* from Yunnan, China, is described based on morphological and genetic data. The new species can be separated from all other species of the genus by having a different shape of the black eye patch, a different coloration of the postorbital and occipital spines and nuchal crest, and a different color of the gular pouch. Genetically, uncorrected sequence divergences of COI between the new species and investigated congeners ranged from 16.12% to 24.11%. The conservation status of the new species is also discussed.

## Introduction

The genus *Acanthosaura* (Gray, 1831) occurs from northeastern India and southern China through Vietnam, Laos, Myanmar, Thailand, Cambodia, the Malay Peninsula and adjacent archipelagos to Sumatra, and the Anambas and Natunas archipelagos (Smith 1935; Taylor 1963; Manthey and Grossmann 1997; Leong et al. 2002; Grismer et al. 2006, 2008a, 2008b, 2011; Manthey 2008; Wood et al. 2010; Uetz et al. 2020). To date, the genus contains 15 species: *A. armata* (Hardwicke & Gray, 1827); *A. lepidogaster* (Cuvier, 1829); *A. capra* Günther, 1861; *A. coronata* Günther, 1861; *A. crucigera* Boulenger, 1885; *A. nataliae* Orlov, Truong & Sang, 2006; *A. bintangensis* Wood, Grismer, Grismer, Ahmad, Onn & Bauer, 2009; *A. titiwangsaensis* Wood, Grismer, Grismer, Ahmad, Onn & Bauer, 2009; *A. cardamomensis* Wood, Grismer, Grismer, Neang, Chav & Holden, 2010; *A. brachypoda* Ananjeva, Orlov, Nguyen & Ryabov, 2011; *A. phuketensis* Pauwels, Sumontha, Kunya, Nitikul, Samphanthamit, Wood & Grismer, 2015; *A. murphyi* Nguyen, Do, Hoang, Nguyen, Mccormack, Nguyen, Orlov, Nguyen & Nguyen, 2018; *A. phongdienensis* Nguyen, Jin, Vo, Nguyen, Zhou, Che, Murphy & Zhang, 2019; *A. tongbiguanensis* Liu & Rao, 2019; and *A. aurantiacrista* Trivalairat, Kunya, Chanhome, Sumontha, Vasaruchapong, Chomngam & Chiangkul, 2020. Most species were described recently, and the diversity of this genus may still be underestimated.

During fieldwork in Honghe Autonomous Prefecture, Yunnan Province, China, some lizards were collected. They had medium-sized bodies, postorbital and occipital spines, a black nuchal collar, and black eye patches, so it can be judged that they belong to the genus *Acanthosaura* (Gray 1831; Zhao et al. 1999; Manthey 2008; Yang and Rao 2008). Morphological and molecular data showed that this population is clearly distinct from all other named species, and it is described and illustrated herein.

## Materials and methods

Specimens were collected from Jianshui County, Honghe Autonomous Prefecture, Yunnan Province, China. Photographs were taken to document color pattern in life prior to euthanization. Liver and muscle tissues were stored in 99% ethanol and lizards were preserved in 75% ethanol. Specimens were deposited at Kunming Natural History Museum of Zoology, Kunming Institute of Zoology, Chinese Academy of Sciences (**KIZ**).

Molecular data were generated for three specimens and available homologous sequences obtained from GenBank; all new sequences have been deposited in GenBank. The agamid *Calotes versicolor* (Daudin, 1802) was used as the outgroup based on Kalyabina-Hauf et al. (2004) and Ananjeva et al. (2008). All the GenBank accession numbers for taxa used in the genetic study can be found in Table 1. Total genomic DNA was extracted from liver tissue stored in 99% ethanol. The mitochondrial DNA gene encoding cytochrome c oxidase subunit I (COI) was sequenced. DNA extraction from tissue samples was done by using proteinase K and the standard three-step phenol/chloroform procedure (Sambrook et al. 1989). PCR was performed using primers Chmf4, 5'-TYTCWACWAAYCAYAAAGAYATCGG-3' and Chmr4, 5'-ACYTCRGGRTGRCCRAARAATCA-3' (Che et al. 2012) in a 25 μl volume with an initial step of 95 °C for 4 min followed by 35 steps of 94 °C for 1 min, 46 °C for 1 min, and 72 °C for 1 min. A final extension involved 72 °C for 10 min. We used a ratio of 0.55 H_2_O : 0.30 ExoI : 0.15 SAP to clean the PCR product (Hanke and Wink 1994). Amplified COI fragments were sequenced in both directions using an ABI PRISM 3730 Automated DNA Sequencer (Applied Biosystems) following the manufacturer’s protocol.

**Table 1.** Specimens, localities, and COI GenBank accession numbers of *Acanthosaura* and outgroup used in this study.

Species	Locality	Voucher no.	GenBank no.
*Acanthosaura armata*	–	NSMT-H4595	AB266452
*Acanthosaura brachypoda*	Sa Pa, Lao Cai, Vietnam	ROM38118	MK695182
	Sa Pa, Lao Cai, Vietnam	ROM38119	MK695183
	Sa Pa, Lao Cai, Vietnam	ROM38120	MK695184
	Sa Pa, Lao Cai, Vietnam	ROM38123	MK695185
*Acanthosaura capra*	Bu Gia Map NP, Binh Phuoc, Vietnam	BGM01	MK239022
*Acanthosaura coronata*	Bu Gia Map, Binh Phuoc, Vietnam	KIZ47	MK695186
	Bu Gia Map, Binh Phuoc, Vietnam	KIZ48	MK695187
	Bu Gia Map, Binh Phuoc, Vietnam	KIZ97	MK695188
	Cat Tien, Dong Nai, Vietnam	ROM42240	MK695189
	Cat Tien, Dong Nai, Vietnam	ROM42241	MK695190
Acanthosaura cf. lepidogaster	Na Hang, Tuyen Quang, Vietnam	ROM30505	MK695191
	Na Hang, Tuyen Quang, Vietnam	ROM30507	MK695192
	Quang Thanh, Cao Bang, Vietnam	ROM30677	MK695193
	Quang Thanh, Cao Bang, Vietnam	ROM30680	MK695194
	Tam Dao, Vinh Phuc, Vietnam	ROM30712	MK695195
	Tam Dao, Vinh Phuc, Vietnam	ROM30715	MK695196
	Chi Linh, Hai Duong, Vietnam	ROM31954	MK695199
*Acanthosaura murphyi*	Hon Ba NR, Khanh Hoa, Vietnam	ITBCZ3533	MK239026
	Deo Ca Forest, Phu Yen, Vietnam	ITBCZ4603	MK239025
	Deo Ca Forest, Phu Yen, Vietnam	PYU147	MK239027
*Acanthosaura nataliae*	Tram Lap, Gia Lai, Vietnam	ROM30629	MK695202
	Tram Lap, Gia Lai, Vietnam	ROM30631	MK695203
	Tram Lap, Gia Lai, Vietnam	ROM30632	MK695204
	Tram Lap, Gia Lai, Vietnam	ITBCZ4994	MK239023
	Tram Lap, Gia Lai, Vietnam	ITBCZ5057	MK239024
*Acanthosaura phongdienensis*	Phong Dien, Thua Thien-Hue, Vietnam	ITBCZ6831	MK695205
	Phong Dien, Thua Thien-Hue, Vietnam	ROM48013	MK695206
	Phong Dien, Thua Thien-Hue, Vietnam	KIZ10657	MK695207
	Phong Dien, Thua Thien-Hue, Vietnam	ITBCZ6830	MK695208
	Phong Dien, Thua Thien-Hue, Vietnam	KIZ10695	MK695209
	Phong Dien, Thua Thien-Hue, Vietnam	KIZ10697	MK695210
Acanthosaura cf. phuketensis	Tanintharyi, Myanmar	USNM587019	MG935416
*Acanthosaura liui* sp. nov.	Jianshui, Honghe, Yunnan, China	KIZL2020003	MT769763
	Jianshui, Honghe, Yunnan, China	KIZL2020004	MT769764
	Jianshui, Honghe, Yunnan, China	KIZL2020005	MT769765
	Jianshui, Honghe, Yunnan, China	KIZL2020006	MT769766
	Jianshui, Honghe, Yunnan, China	KIZL2020007	MT769767
*Calotes versicolor*	Ta Kou, Binh Thuan, Vietnam	ITBCZ1034	MK695212

Sequences were aligned using ClustalW (Thompson et al. 1994) integrated in MEGA 7 (Tamura et al. 2013) with default parameters. Pairwise distances between species were calculated in MEGA 7 (Tamura et al. 2013). The substitution model GTR was selected in MODELTEST v3.7 (Posada and Crandall 1998). Bayesian inference (BI) was performed in MrBayes 3.2.6 (Ronquist et al. 2012) based on the selected substitution model. Two runs were performed simultaneously with four Markov chains starting from random tree. The chains were run for 1,000,000 generations and sampled every 100 generations. The first 25% of the sampled trees was discarded as burn-in after the standard deviation of split frequencies of the two runs was less than a value of 0.01, and then the remaining trees were used to create a 50% majority-rule consensus tree and to estimate Bayesian posterior probabilities (BPPs). Maximum likelihood (ML) analysis was performed in RaxmlGUI 1.5 (Silvestro and Michalak 2012), and nodal support was estimated by 1,000 rapid bootstrap replicates. Maximum Parsimony (MP) analyses were done using TNT (Goloboff et al. 2008), confidence in tree topology was tested by 1000 generations for non-parametric bootstraps (Felsenstein 1985).

Meristic and mensural characters were noted for each specimen of the type series (Table 3). Measurements were taken to the nearest 0.1 mm with digital calipers. Paired measurements were made on the left side (Wood et al. 2009, 2010; Ananjeva et al. 2011). Paired meristic characters were given as left/right. The methodology of measurements and meristic counts followed Liu et al. (2019):

**BEP** presence (1) or absence (0) of a black eye patch;

**CS** number of canthus rostralis-supraciliary scales, counted from the nasal scale to the posterior end of the ridge at the posterior margin of the orbit;

**DIAS** length of the diastema, measured from the posterior end of the nuchal crest to the anterior end of the dorsal crest;

**DS** maximum length of the largest spine in the dorsal crest, measured from the base to the tip;

**DSL** longest dorsal scale, measured at the base below the dorsal crest;

**ESBO** presence (1) or absence (0) of elliptical scales below the orbit;

**EYE** eye diameter, measured from the posterior to the anterior edge of the eye;

**FI** number of subdigital lamellae on the fourth finger;

**FOREL** forelimb length, measured from axilla to the proximal edge of the palmar region;

**GP** size of gular pouch, scored as absent (0), small (1), medium (2), large (3), or very large (4);

**HD** maximum head height, measured across the parietal region;

**HINDL** hindlimb length, measured from groin to the proximal edge of the plantar region;

**HL** head length, measured from posterior edge of the lower jaw to the tip of the snout;

**HW** head width, maximum head width, the width at the level of the tympanum;

**INFRAL** number of infralabials;

**LKP** presence (1) or absence (0) of light knee patch;

**MH** mental height;

**MW** mental width;

**ND** presence (1) or absence (0) of a black, diamond shaped, nuchal collar;

**NR** number of scales between the nasal and the rostral;

**NSL** maximum length of the largest spine in the nuchal crest measured from the base to the tip;

**NN** number of nuchal crest spines;

**NSSOS** number of scales surrounding the occipital spine;

**NSSPS** number of scales surrounding the postorbital spine;

**OF** presence (1) or absence (0) of oblique humeral fold;

**ORBIT** orbit diameter, measured from the posterior to the anterior edge of the orbit;

**OS** occipital spine length, measured from the base to the tip of the spine;

**PM** number of scales bordering the mental;

**PS** postorbital spine length, measured from the base to the tip of the spine;

**RH** rostral height;

**RS** number of scales bordering the rostral;

**RW** rostral width;

**SL** snout length, measured from the anterior edge of the orbit to the tip of the snout;

**SUPRAL** number of supralabials;

**SVL** snout-vent length, measured from the tip of the snout to the tip of the vent;

**TL** tail length, measured from the posterior margin of the vent to the tip of the tail;

**TBW** tail base width, maximum width at tail base;

**TD** tympanum diameter, measured horizontally from the anterior to the posterior border of the tympanum;

**TN** presence (1) or absence (0) of scales on tympanum;

**TO** subdigital lamellae on the fourth toe;

**VENT** number of ventral scales, counted at the midline from the anterior edge of the shoulders to the edge of the vent;

**WNC** maximum width of the spines in the nuchal crest, measured at the base;

**WDS** maximum width of the largest dorsal scale below the dorsal crest, measured at the base;

**YAS** presence (1) or absence (0) of a Y-shaped arrangement of enlarged scales on the snout.

Comparative morphological data were taken from original descriptions and subsequent studies (Hardwicke and Gray 1827; Cuvier 1829; Günther 1861, 1864; Boulenger 1885, 1900; Werner 1904; Vogt 1914; Lönnberg 1916; Smith 1935; Taylor 1963; Orlov et al. 2006; Ananjeva et al. 2008, 2011; Wood et al. 2009, 2010; Pauwels et al. 2015; Nguyen et al. 2018; Liu et al. 2019; Nguyen et al. 2019; Trivalairat et al. 2020).

## Results

**Figure 1.**
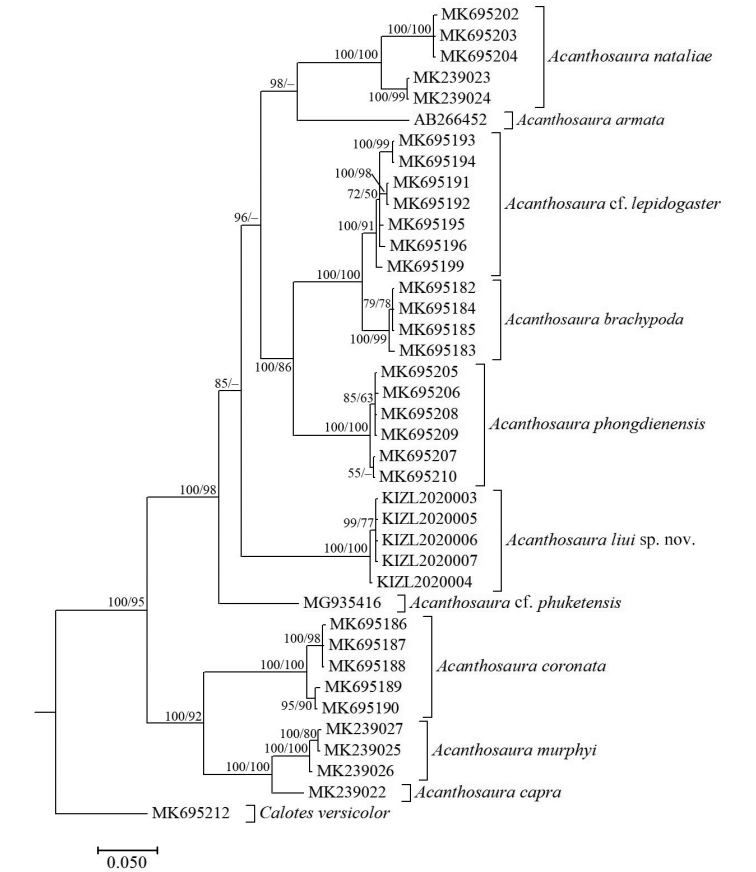
Bayesian and Maximum likelihood phylogram of investigated members of *Acanthosaura* inferred from COI gene. Numbers before slashes indicate Bayesian posterior probabilities and numbers after slashes indicate bootstrap support for Maximum likelihood analyses. The symbol “–” represents value below 50.

**Figure 2.**
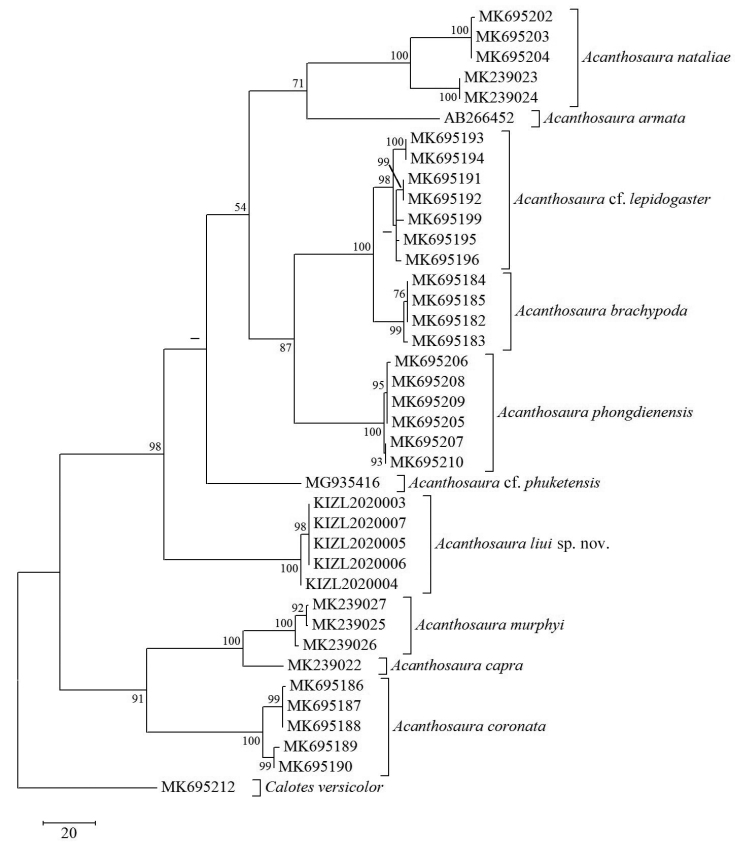
Maximum Parsimony phylogram of investigated members of *Acanthosaura* inferred from COI gene. Numbers beside the nodes indicate bootstrap support for Maximum Parsimony analyses. The symbol “–” represents value below 50.

The obtained sequence alignment is 610 bp in length. BI and ML analyses showed the same result (Fig. 1) and MP analyses showed a similar result (Fig. 2) to BI and ML. All results indicated that the new species is a separate taxon in the genus *Acanthosaura* with weak (BI and ML) or strong support (MP). Uncorrected *p*-distances (Table 2) between investigated congeners ranged from 4.23% to 24.94%. The new species differed from investigated congeners ranging from 16.12% to 24.11% in *p*-distances.

**Table 2.** Average *p*-distances (%) among some members of *Acanthosaura* and outgroup calculated from COI gene sequences.

Species		1	2	3	4	5	6	7	8	9	10
**1**	*Acanthosaura armata*										
**2**	*Acanthosaura brachypoda*	18.16									
**3**	*Acanthosaura capra*	20.60	21.36								
**4**	*Acanthosaura coronata*	23.09	23.03	16.87							
**5**	Acanthosaura cf. lepidogaster	19.01	4.23	21.26	21.97						
**6**	*Acanthosaura murphyi*	20.78	22.08	6.39	17.25	21.15					
**7**	*Acanthosaura nataliae*	18.26	17.84	24.94	24.51	18.06	24.63				
**8**	*Acanthosaura phongdienensis*	17.47	13.63	21.91	21.65	12.94	21.73	18.61			
**9**	Acanthosaura cf. phuketensis	18.47	16.56	21.31	20.67	15.71	20.60	16.87	16.04		
**10**	*Acanthosaura liui* sp. nov.	19.67	19.26	22.56	24.11	17.51	22.51	20.13	17.74	16.12	
**11**	*Calotes versicolor*	23.62	23.89	22.91	23.48	24.71	23.33	27.74	24.96	23.45	25.09

### Systematics

#### Acanthosaura liui sp. nov.

Taxon classificationAnimaliaSquamataAgamidae

1C1F3E1F-5922-5C1F-9528-E84AD9F1F59F

http://zoobank.org/8E1E9E67-BB71-41CA-9997-CEB3A3B63955

**Figure 3.**
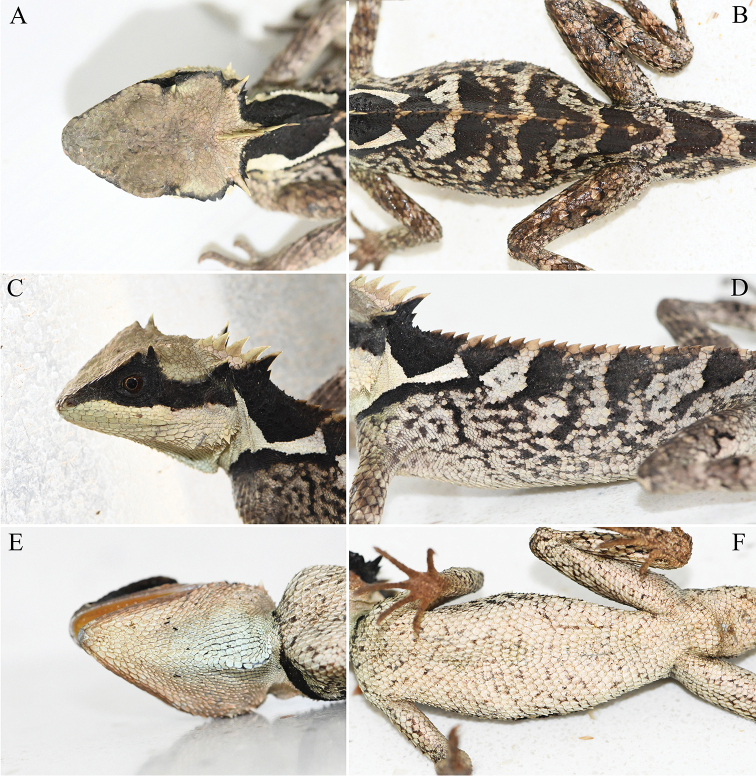
Adult male holotype (KIZL2020001) of *Acanthosaura liui* sp. nov. in life. **A** dorsal view of the head **B** dorsal view of the body **C** lateral view of the head **D** lateral view of the body **E** ventral view of the head **F** ventral view of the body.

**Figure 4.**
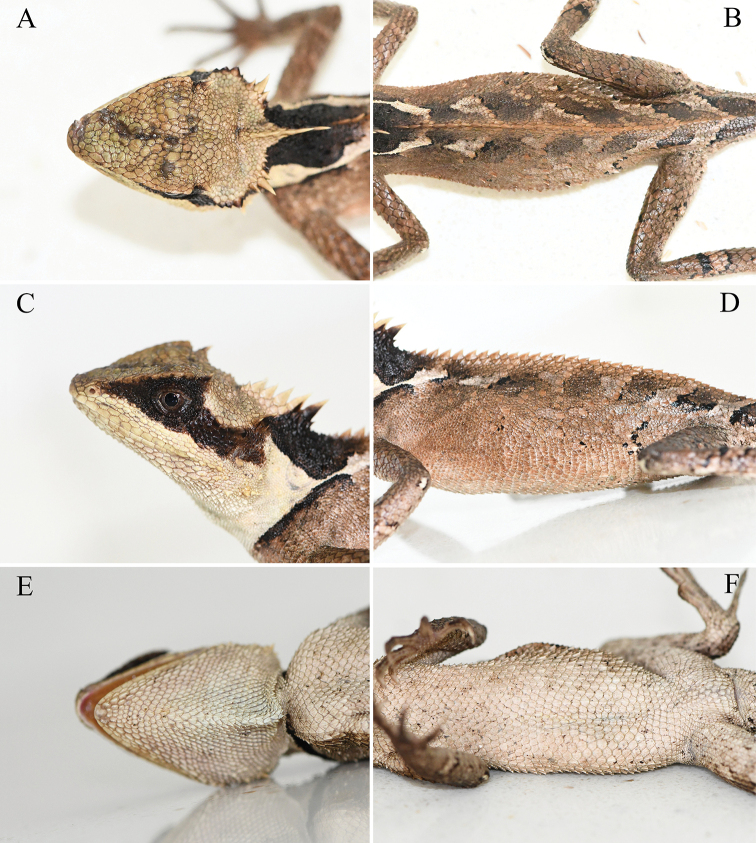
Adult female paratype (KIZL2020002) of *Acanthosaura liui* sp. nov. in life. **A** dorsal view of the head **B** dorsal view of the body **C** lateral view of the head **D** lateral view of the body **E** ventral view of the head **F** ventral view of the body.

**Figure 5.**
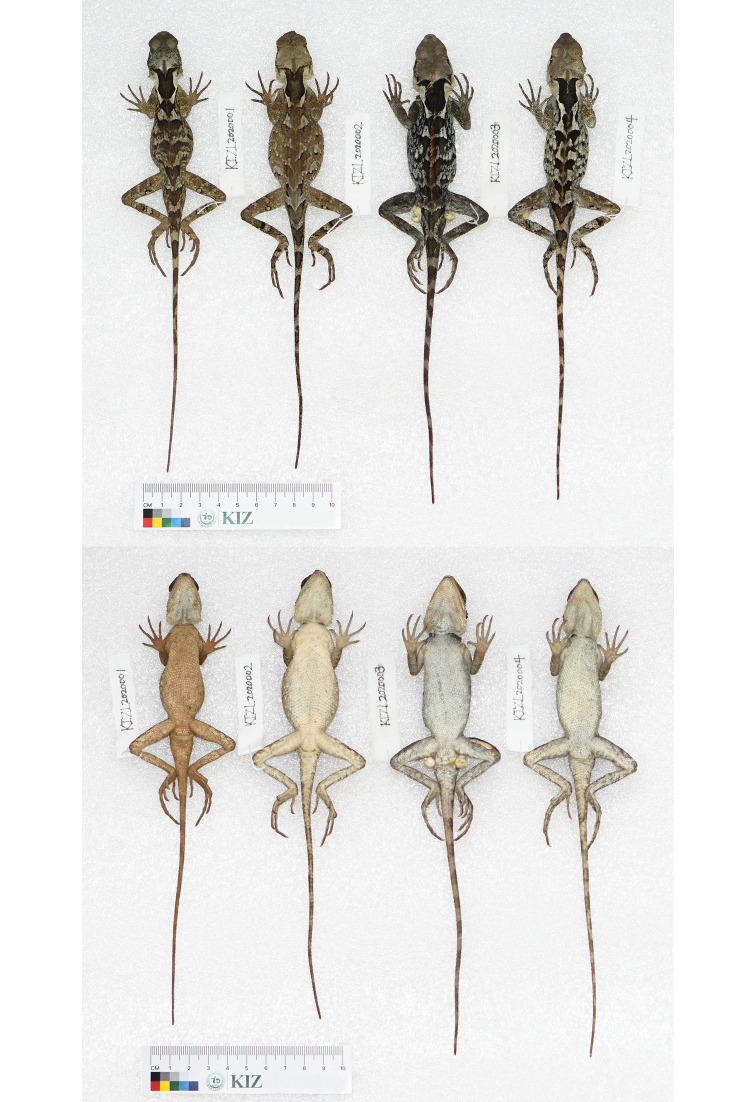
Dorsal view (top) and ventral view (bottom) of type series of *Acanthosaura liui* sp. nov. in preservative. From left to right: male holotype (KIZL2020001), female paratype (KIZL2020002), male paratype (KIZL2020003), male paratype (KIZL2020004).

Figures 3 , 4 , 5 , 8

##### Holotype.

China • ♂; Yunnan, Jianshui; 23°20'24"N, 102°44'28"E; 1540 m; 2 May. 2020; Shuo Liu leg.; KIZL2020001.

##### Paratypes.

China • 1 ♀; the same locality as the holotype; 16 May. 2020; Shuo Liu leg.; KIZL2020002 • 2 ♂; the same locality as the holotype; 17 May. 2020; Shuo Liu leg.; KIZL2020003 and KIZL2020004.

##### Etymology.

The specific epithet is dedicated to the renowned herpetologist Prof. Chengzhao Liu (30.9.1900–9.4.1976), in recognition of his great contributions to herpetological research in China.

##### Diagnosis.

Body size medium (SVL 85.1–95.9 mm), postorbital and occipital spines present; relatively developed gular pouch; scales on flanks randomly intermixed with medium and large scales; nuchal crest gradually developed posteriorly, first nuchal crest spine shortest and last nuchal crest spine longest; diastema between nuchal and dorsal crests present; dorsal crest underdeveloped, composed of enlarged, pointed scales beginning at shoulder region and decreasing regularly in size; tail 1.47–1.77× SVL; number of subdigital lamellae on the fourth finger 16–18 and the fourth toe 22–25; black nuchal collar present; black eye patch extending from nostril through orbit posteriorly and downwards through tympanum and then posteriorly and upwards meeting diamond-shaped black nuchal collar on nape; black oblique folds anterior to forelimb insertions present and not meeting black nuchal collar; anterior nuchal crest spines and dorsal sides of postorbital and occipital spines light colored, posterior nuchal crest spines and ventral sides of postorbital and occipital spines black; gular pouch light blue; tongue and inside of mouth yellow.

##### Description of the holotype.

Adult male. SVL 85.1 mm. TL 150.6 mm, tail complete. Head length 26.7 mm; head moderately long (HL/SVL 31%), somewhat narrow (HW/SVL 22%), not tall (HD/HL 57%), triangular in dorsal and lateral profile. Snout short (SL/HL 40%), a Y-shaped arrangement of enlarged scales on the snout; interorbital and frontal regions and rostrum wide. Canthus rostralis prominent, forming a large projecting shelf extending above eye, composed of 12/13 enlarged scales; shelf terminates with a notch anterior to the postorbital spine. Nasal concave, nostrils surrounded by a circular scale. Eye relatively large (EYE/HL 22%), orbit very large (ORBIT/HL 31%). Prefrontal and frontal scales slightly keeled and larger than scales between supralabials; scales on occiput weakly keeled. Moderately elongate epidermal spine above posterior margin of eye, straight, surrounded by 5/5 enlarged scales. A notch present between the supraciliary edge and postorbital spine. Moderately elongate epidermal spine on occipital region, straight, surrounded by a rosette of 5/5 short spiny scales. A row of enlarged conical scales present between orbit and occipital spine; a few elongate conical scales present on the posterior of lower jaw. Tympanum exposed, oblong, surrounded by small scales. Supralabials 10/11, rectangular, scales in center of series largest; infralabials 10/11, rectangular, scales in center of series largest; rostral oblong, eight scales contacting the rostral; mental squarish above, becoming triangular below, larger than first pair of infralabials, six scales contacting the mental; gulars sharply keeled and spinose. Dewlap extensible, gular pouch relatively developed. Nuchal crest composed of five elongate, lanceolate, laterally compressed scales bordered on each side by one row of enlarged, spinose scales; nuchal crest gradually developed posteriorly, the first nuchal crest spine shortest and the last nuchal crest spine longest; nuchal crest followed by a diastema at base of nape. Dorsal body crest underdeveloped, extending from posterior margin of diastema onto base of tail; vertebral crest composed of enlarged, epidermal, laterally compressed, spinose scales; vertebral crest tapers slightly to base of tail, then fades progressively. Body slightly short, triangular in cross-section. Dorsal scales small, mixed with large scales indistinctly arranged in diagonal rows, keels projecting posterior wards; scales of pectoral region and abdomen larger than dorsal scales, keeled, more or less arranged in transverse rows; keeled scales anterior to vent not enlarged. Limbs relatively long (FOREL/SVL 42%, HINDL/SVL 60%); dorsal and ventral scales of forelimbs keeled, spinose, approximately the same size. Five digits on manus; subdigital scales keeled, subdigital lamellae under fourth finger 17/17. Scales of hind limbs keeled and spinose; postfemoral scales small, interspersed with larger spinose scales. Five digits on pes; subdigital scales keeled, subdigital lamellae under fourth toe 25/25. Tail length 1.77 times SVL, tail covered with keeled spinose scales, keels on subcaudals directed posteriorly; subcaudals much longer than supracaudals; base of tail 12.3 mm wide.

##### Color of holotype in life.

Dorsal surface of head light brownish grey; black eye patch extending from nostril through orbit posteriorly and downwards through tympanum and then posteriorly and upwards meeting the diamond-shaped black nuchal collar on nape; upper and lower lips white, same as color of lateral and ventral sides of neck; iris reddish brown; black nuchal collar does not reach black oblique folds anterior to fore limb insertions; gular pouch light blue; dorsal sides of postorbital and occipital spines same color as dorsal surface of head, ventral sides of postorbital and occipital spines black, first four nuchal crest spines almost white, last nuchal crest spine black; tongue and inside of mouth yellow; a few almost symmetrical large black and greyish white speckles on both sides of dorsal crest, many small black and light purplish grey speckles on lateral sides of body; dorsal surface of limbs brown with indistinct lightly colored transverse stripes, light patches on elbows and knees; ventral sides of limbs and body white with many small brown spots; brownish black and greyish white rings on tail. However, these lizards can change their body color within limits like most other members of this genus.

##### Color of holotype in preservative.

The coloration in preservative resembles that in life. Light blue on gular region faded; dorsal surface of head and upper and lower lips became darker; spots on belly became indistinct.

##### Variation.

Morphometric and meristic data for the type series are provided in Table 3. The female paratype resembles the holotype in most aspects except that it has a larger body size and a shorter tail, a darker dorsal ground-color of the body, and with its penultimate nuchal crest spine being half black; the light blue color on gular region is indistinct, and the base of the tail is not thickened. The two male paratypes resemble the holotype in most aspects except that they have bigger body sizes and relatively shorter tails, no spot on the ventral sides of their bodies, and both have seven nuchal crest spines.

**Table 3.** Morphometrical (in mm) and meristic data for the type series of *Acanthosaura liui* sp. nov. For character abbreviations see material and methods. Paired meristic characters are given left/right.

	Holotype KIZL2020001 Adult male	Paratype KIZL2020002 Adult female	Paratype KIZL2020003 Adult male	Paratype KIZL2020004 Adult male	Range	Mean
SVL	85.1	94.8	95.9	94.1	85.1–95.9	92.5
TL	150.6	139.3	155.1	155.3	139.3–155.3	150.1
TL/SVL	1.77	1.47	1.62	1.65	1.47–1.77	1.63
TBW	12.3	10.6	13.9	13.7	10.6–13.9	12.6
HL	26.7	29.2	30.0	28.7	26.7–30.0	28.7
HW	18.3	19.5	22.4	19.8	18.3–22.4	20.0
HD	15.1	15.8	17.3	16.7	15.1–17.3	16.2
SL	10.7	11.2	11.3	10.3	10.3–11.3	10.9
ORBIT	8.4	8.8	8.6	8.9	8.4–8.9	8.7
EYE	5.8	6.4	5.9	6.1	5.8–6.4	6.1
TD	2.9	3.8	3.4	3.5	2.9–3.8	3.4
TD/HD	0.19	0.24	0.20	0.21	0.19–0.24	0.21
TN	0	0	0	0	0	0
PS	3.0	2.5	2.1	3.2	2.1–3.2	2.7
PS/HL	0.11	0.09	0.07	0.11	0.07–0.11	0.10
NSSPS	5/5	6/5	5/5	5/6	5–6	5
NSL	5.2	5.1	7.1	4.9	4.9–7.1	5.6
NSL/HL	0.19	0.17	0.24	0.17	0.17–0.24	0.19
DS	2.7	3.2	3.7	3.5	2.7–3.7	3.3
DS/HL	0.10	0.11	0.12	0.12	0.10–0.12	0.11
NN	5	5	7	7	5–7	6
DSL	1.7	1.9	1.9	1.6	1.6–1.9	1.8
WNC	1.1	1.2	1.3	1.1	1.1–1.3	1.2
WDS	0.9	1.0	0.9	1.0	0.9–1.0	1.0
DIAS	3.5	4.7	4.7	4.2	3.5–4.7	4.3
DIAS/SVL	0.04	0.05	0.05	0.04	0.04–0.05	0.05
FOREL	35.8	37.0	36.8	36.2	35.8–37.0	36.5
HINDL	50.7	50.8	52.6	51.4	50.7–52.6	51.4
SUPRAL	10/11	11/10	13/11	12/11	10–13	11
INFRAL	10/11	11/10	11/10	11/11	10–11	11
VENT	53	54	56	52	52–56	54
FI	17/17	16/17	18/17	17/18	16–18	17
TO	25/25	22/24	23/23	24//24	22–25	24
OS	3.8	3.6	4.8	4.7	3.6–4.8	4.2
OS/HL	0.14	0.12	0.16	0.16	0.12–0.16	0.15
NSSOS	5/5	6/5	5/5	4/4	4–6	5
CS	12/13	12/13	12/13	12/13	12–13	13
RW	3.4	3.7	4.2	3.8	3.4–4.2	3.8
RH	1.4	1.6	1.5	1.9	1.4–1.9	1.6
RS	8	9	9	8	8–9	9
NR	1/1	2/2	2/2	2/1	1–2	2
MW	1.8	2.2	2.4	2.3	1.8–2.4	2.2
MH	1.7	2.0	1.8	2.3	1.7–2.3	2.0
PM	6	6	6	5	5–6	6
YAS	1	1	1	1	1	1
ND	1	1	1	1	1	1
LKP	1	1	1	1	1	1
BEP	1	1	1	1	1	1
ESBO	0	0	0	0	0	0
GP	3	2	3	3	2–3	3
OF	1	1	1	1	1	1

##### Distribution.

After our extensive field investigation, *Acanthosaura liui* sp. nov. is only recorded in Jianshui County, Gejiu City, and Shiping County, Honghe Autonomous Prefecture, Yunnan, China.

##### Natural history.

The specimens of *Acanthosaura liui* sp. nov. were all found at night while they were asleep on trees beside rivers. At the type locality, we found seven other species of reptiles, namely *Hebius atemporale* (Bourret, 1934), *Ophiophagus hannah* (Cantor, 1836), *Oreocryptophis porphyraceus* (Cantor, 1839), *Pareas margaritophorus* (Jan, 1866), *Ptyas major* (Günther, 1858), *P. nigromarginata* (Blyth, 1854), and *Sphenomorphus indicus* (Gray, 1853), and three species of amphibians, namely *Hyla annectans* (Jerdon, 1870), *Nidirana pleuraden* (Boulenger, 1904), and *Polypedates megacephalus* Hallowell, 1861.

##### Comparisons.

*Acanthosaura liui* sp. nov. differs from *A. capra*, *A. murphyi*, and *A. nataliae* by the presence of occipital spines (vs absent in the latter species).

*Acanthosaura liui* sp. nov. differs from *A. armata*, *A. aurantiacrista*, *A. cardamomensis*, and *A. phuketensis* by having much shorter postorbital, occipital, nuchal crest, and dorsal crest spines (nuchal and dorsal spines <10 mm in the new species vs >10 mm in the latter species).

*Acanthosaura liui* sp. nov. differs from *A. bintangensis* by the presence of a light knee patch (vs absent in the latter species), the absence of an enlarged row of keeled scales below orbit (vs present), the absence of large yellow spots edged in blackish brown arranged on body and base of tail (vs present); the black eye patch in *Acanthosaura liui* sp. nov. extends backwards and downwards through the tympanum and continues backwards and upwards to reach the black nuchal collar while it never extends onto the head side in *A. bintangensis*.

*Acanthosaura brachypoda* is known only from the single holotype (Ananjeva et al. 2011), so we only compared the female *Acanthosaura liui* sp. nov. with the characters of the female holotype of *A. brachypoda*. *Acanthosaura liui* sp. nov. differs from *A. brachypoda* by the presence of gular pouch (vs absent). *Acanthosaura liui* sp. nov. does not have pairs of transverse creamy spots along both sides of spine forming a symmetrical pattern present as in *A. brachypoda*. The nuchal crest is gradually developed posteriorly, the first nuchal crest spine is shortest, and the last nuchal crest spine is longest, while the nuchal crest is composed of two enlarged terminal (anterior and posterior) scales and much smaller scales between them in *A. brachypoda*.

*Acanthosaura liui* sp. nov. differs from *A. coronata* by the presence of obvious postorbital spines, occipital spines, nuchal and dorsal crests (vs absent or not obvious), a diastema between nuchal crest and dorsal crest (vs a continuous nuchal and dorsal crest), the presence of a black nuchal collar (vs absent), the presence of a black eye patch (vs absent), and the presence of a gular pouch (vs absent).

*Acanthosaura liui* sp. nov. differs from *A. crucigera* (and the synonymous *A. horrescens* Lönnberg, 1916) by in that its black eye patch extends backwards and downwards through the tympanum and backwards and upwards to the black nuchal collar, while in *A. crucigera* the black eye patch only extends to the anterior edge of the tympanum. In the new species the black nuchal collar does not extend downwards to reach the black oblique humeral fold, while in *A. crucigera* the black nuchal collar extends downwards to reach the black oblique humeral fold.

*Acanthosaura lepidogaster* is a complex of cryptic species and has a very wide distribution including Vietnam, Thailand, Myanmar, Laos, and southern China, and its type locality is uncertain (Ananjeva et al. 2008, 2011; Nguyen et al. 2019). So, except for the type specimens, other populations of this complex are referred to as *A. lepidogaster* sensu lato. Thus, we compared *Acanthosaura liui* sp. nov. with the the original description (Cuvier 1829) and photographs of the type specimen (MNHN 5076) (Ananjeva et al. 2011) of *A. lepidogaster* sensu stricto. *Acanthosaura liui* sp. nov. differs from *A. lepidogaster* in having relatively fewer nuchal crest spines and a relatively larger gular pouch. The nuchal crest is gradually developed posteriorly in the new species; from the second nuchal crest spine, each spine is obviously longer than the previous one, while nuchal crest spines are almost the same length except for the first one in *A. lepidogaster*. The distance between the two occipital spines are almost equal to the distance between the two postorbital spines in the new species, while the distance between the two occipital spines are obviously less than the distance between the two postorbital spines in *A. lepidogaster*. Three significantly projecting conical scales above the tympanum make the head appear distinct triangular from above in the new species, while in *A. lepidogaster* there are some slightly projecting conical scales above the tympanum, but the head is not distinctly triangular from above. For *A. braueri* Vogt, 1914, *A. fruhstorferi* Werner, 1904, *A. hainanensis* Boulenger, 1900, and *A. lamnidentata* Boulenger, 1885, which have been synonymized with *A. lepidogaster*, some of them may represent valid species in the *A. lepidogaster* complex and need re-evaluation. According to the literature (Boulenger 1885, 1900; Werner 1904; Vogt 1914; Hallermann 2000), *Acanthosaura liui* sp. nov. can be distinguished from all of them by the following characters: occipital spine obviously longer than postorbital spine, gular pouch well developed, dorsal surface of head light colored, and black eye patch extending backwards and downwards through the tympanum and then backwards and upwards to reach the black nuchal collar.

*Acanthosaura liui* sp. nov. differs from *A. phongdienensis* in having a diastema between the nuchal crests and dorsal crests (vs a continuous nuchal and dorsal crest). The black eye patch in *Acanthosaura liui* sp. nov. extends backwards and downwards through the tympanum and then backwards and upwards to reach the black nuchal collar, while in *A. phongdienensis* it extends backwards and upwards above the tympanum to reach the black nuchal collar.

*Acanthosaura liui* sp. nov. differs from *A. titiwangsaensis* by the presence of light knee patch (vs absent), the absence of medium-sized, light orange spots edged with a faded black color on the body and base of the tail (vs present); the black eye patch in *Acanthosaura liui* sp. nov. extends backwards and downwards through the tympanum and continues backwards and upwards to reach the black nuchal collar, while in *A. titiwangsaensis* it is restricted to the orbit and does not extend into the postorbital region.

*Acanthosaura liui* sp. nov. differs from *A. tongbiguanensis* in having shorter postorbital and occipital spines. The black eye patch in *Acanthosaura liui* sp. nov. extends backwards and downwards through the tympanum and continues backwards and upwards to reach the black nuchal collar, while in *A. tongbiguanensis* it extends backward and downward beyond the posterior end of the tympanum but never continues backward and upward to reach the black nuchal collar. The black nuchal collar does not extend downwards to reach the black oblique humeral fold, while in *A. tongbiguanensis* it extends downwards to reach the black oblique humeral fold. The tongue and the inside of the mouth are yellow in *Acanthosaura liui* sp. nov., while they are pink in *A. tongbiguanensis*. The gular region is light blue in the new species, while it is white in *A. tongbiguanensis*.

## Discussion

The sequence with GenBank accession number MG935416 was identified as *Acanthosaura crucigera* by Mulcahy et al. (2018); however, the specimen has very long postorbital spines, occipital spines, nuchal crest spines, and dorsal crest spines (Mulcahy et al. 2018: fig. 4), and most similar to *A. phuketensis*. Additionally, the collection site of this specimen is near Phuket Island, Thailand, the type locality of *A. phuketensis*. Thus, we consider this specimen as A. cf. phuketensis.

Previously, there were two known species of *Acanthosaura* in Yunnan, China: *A. lepidogaster* complex and *A. tongbiguanensis*. *Acanthosaura tongbiguanensis* is only found in Dehong Autonomous Prefecture (Liu et al. 2019), western Yunnan, and *A. lepidogaster* complex occurs in western, southwestern, southern, and southeastern Yunnan (Zhao et al. 1999; Yang and Rao 2008). *Acanthosaura liui* sp. nov. is only found in south-central Yunnan. To date, no overlap between the distributions of the three species was found.

**Figure 6.**
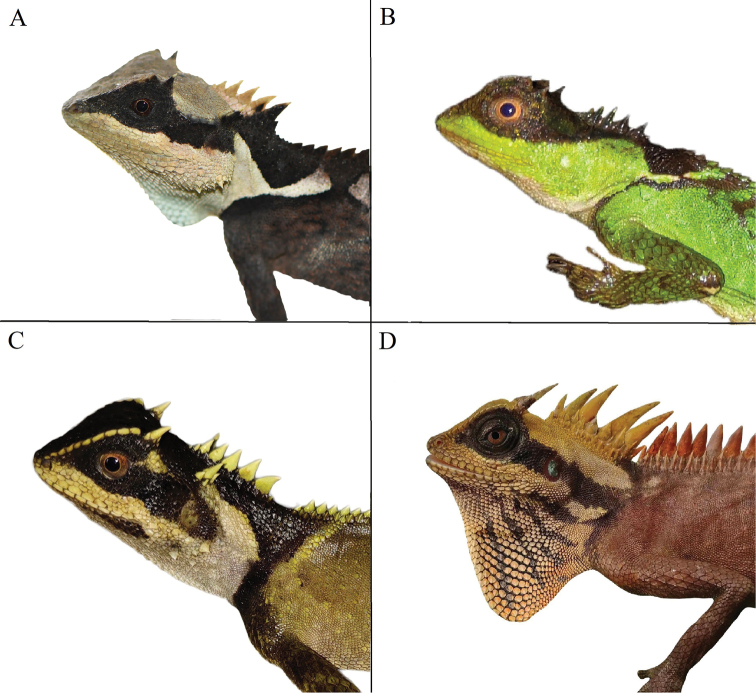
Comparison of four different types of eye patch. **A***Acanthosaura liui* sp. nov. (from Jianshui, Yunnan, China) **B**A. cf. lepidogaster (from Trung Khanh, Cao Bang, Vietnam) **C***A. tongbiguanensis* (from Yingjiang,Yunnan, China) **D***A. nataliae* (from Gia Lai, Vietnam).

**Figure 7.**
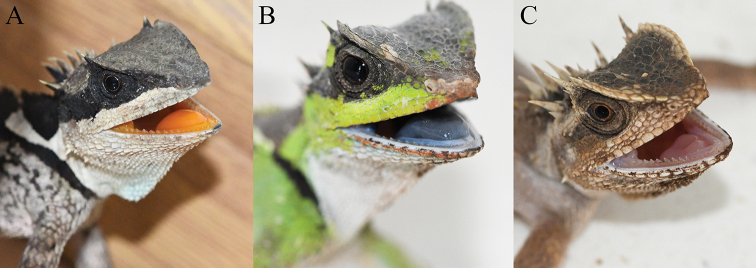
Comparison of the colors of tongue and inside of the mouth. **A***Acanthosaura liui* sp. nov. (from Jianshui, Yunnan, China) **B**A. cf. lepidogaster (from Trung Khanh, Cao Bang, Vietnam) **C***A. tongbiguanensis* (from Yingjiang, Yunnan, China).

**Figure 8.**
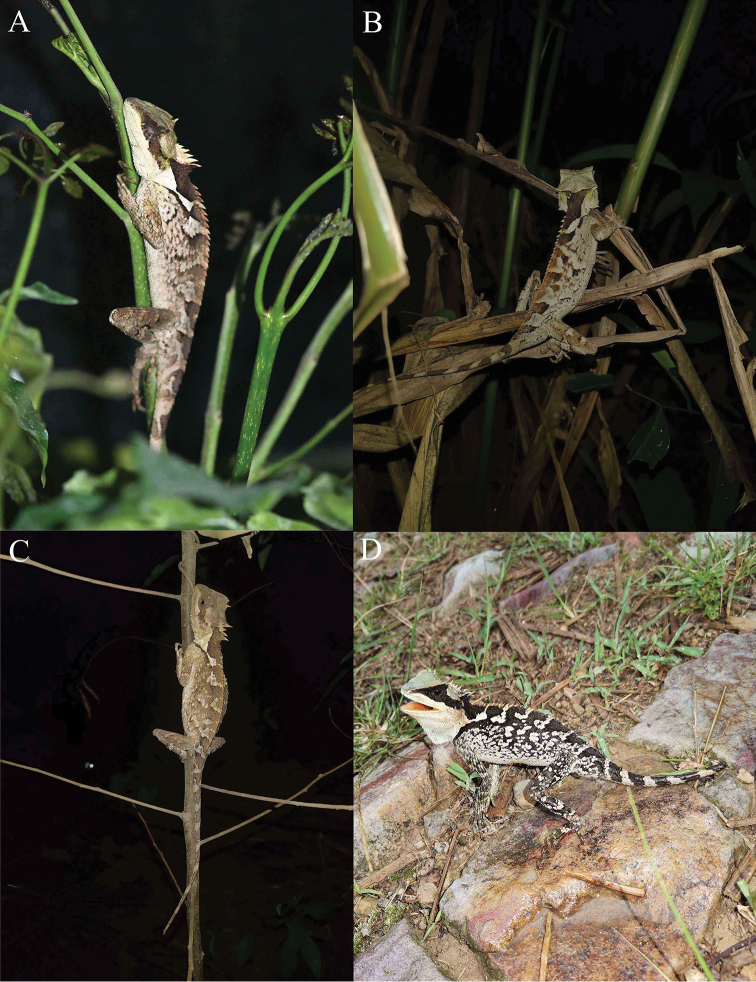
Live *Acanthosaura liui* sp. nov. in the field. **A** an adult male asleep on a branch **B** an adult male asleep on dead leaves **C** an adult female asleep on a branch **D** an adult male on the ground.

In areas where *Acanthosaura liui* sp. nov. occurs, the primordial forest has been severely destroyed and reforested with exotic trees. The forests are now surrounded by farmland and villages, and the habitat is very fragmented. Furthermore, many roads have been built and more roads are being built locally. This is detrimental, as we found that many lizards and other reptiles had died on these roads (Fig. 10). Considering the restricted habitat and distribution of the new species, we suggest International Union for Conservation of Nature status as Endangered. We have suggested to the local forestry department that this endemic species be added to the protected animal lists and that the habitat of this species be protected.

**Figure 9.**
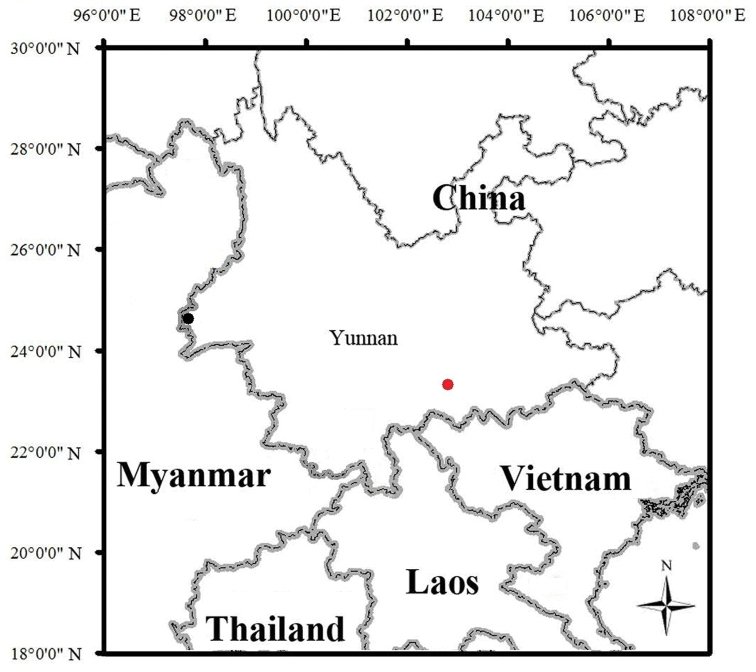
The type locality of *Acanthosaura liui* sp. nov. (red dot), and the type locality of *A. tongbiguanensis* (black dot) in Yunnan Province, China.

**Figure 10.**
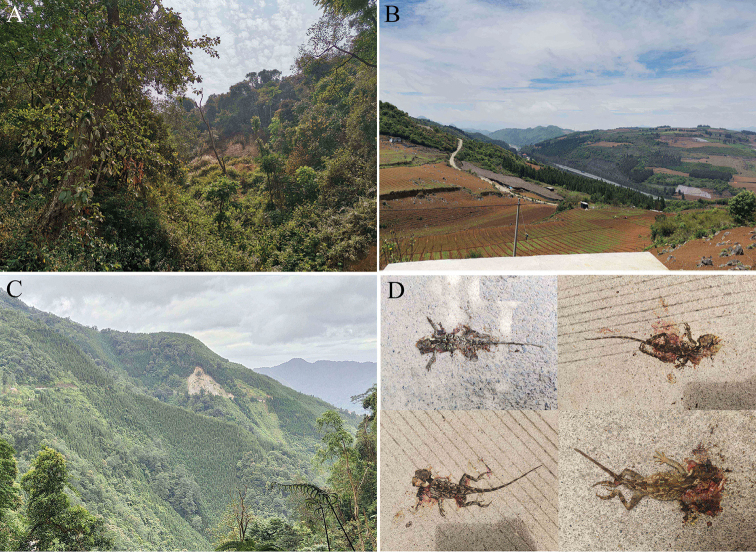
**A** Habitat of *Acanthosaura liui* sp. nov. at the type locality. **B** Large tracts of farmland surrounding the habitat of *Acanthosaura liui* sp. nov. **C** Large tracts of reforestation with exotic trees surrounding the habitat of *Acanthosaura liui* sp. nov. **D** Many individuals of *Acanthosaura liui* sp. nov. died under the wheels on the highways passing through the habitat of *Acanthosaura liui* sp. nov.

## Supplementary Material

XML Treatment for Acanthosaura liui
